# Rare but diverse off-target and somatic mutations found in field and greenhouse grown trees expressing CRISPR/Cas9

**DOI:** 10.3389/fbioe.2024.1412927

**Published:** 2024-06-21

**Authors:** Greg S. Goralogia, Isabella M. Andreatta, Victoria Conrad, Qin Xiong, Kelly J. Vining, Steven H. Strauss

**Affiliations:** ^1^ Department of Forest Ecosystems and Society, Oregon State University, Corvallis, OR, United States; ^2^ Co-Innovation Center for Sustainable Forestry in Southern China, College of Life Science, Nanjing Forestry University, Nanjing, China; ^3^ Department of Horticulture, Oregon State University, Corvallis, OR, United States

**Keywords:** CRISPR, gene editing, off-target editing, *Populus*, *Eucalyptus*, tree biotechnology

## Abstract

**Introduction:** CRISPR gene editing, while highly efficient in creating desired mutations, also has the potential to cause off-target mutations. This risk is especially high in clonally propagated plants, where editing reagents may remain in the genome for long periods of time or in perpetuity. We studied a diverse population of *Populus* and *Eucalyptus* trees that had CRISPR/Cas9-containing transgenes that targeted one or two types of floral development genes, homologs of *LEAFY* and *AGAMOUS*.

**Methods:** Using a targeted sequence approach, we studied approximately 20,000 genomic sites with degenerate sequence homology of up to five base pairs relative to guide RNA (gRNA) target sites. We analyzed those sites in 96 individual tree samples that represented 37 independent insertion events containing one or multiples of six unique gRNAs.

**Results:** We found low rates of off-target mutations, with rates of 1.2 × 10^−9^ in poplar and 3.1 × 10^−10^ in eucalypts, respectively, comparable to that expected due to sexual reproduction. The rates of mutation were highly idiosyncratic among sites and not predicted by sequence similarity to the target sites; a subset of two gRNAs showed off-target editing of four unique genomic sites with up to five mismatches relative to the true target sites, reaching fixation in some gene insertion events and clonal ramets. The location of off-target mutations relative to the PAM site were essentially identical to that seen with on-target CRISPR mutations.

**Discussion:** The low rates observed support many other studies in plants that suggest that the rates of off-target mutagenesis from CRISPR/Cas9 transgenes are negligible; our study extends this conclusion to trees and other long-lived plants where CRISPR/Cas9 transgenes were present in the genome for approximately four years.

## 1 Introduction

Gene editing technologies using site-specific nucleases (SSNs) such as CRISPR/Cas9 has been a transformative method for scientific research and biotechnology (Adli, 2018). Gene editing using wild type CRISPR/Cas systems, most commonly SpCas9 from *Streptococcus pyogenes*, has been widely employed throughout angiosperm plants, most commonly through *Agrobacterium*-mediated stable integration of a Cas/gRNA-containing transgene (Goralogia et al., 2021; Cardi et al., 2023). For most plants, removal of stably integrated transgenes via segregation is the common approach, after which null segregants containing the desired mutations absent transgenes are the starting points for scientific research or biotechnology. Current regulations in the United States permit null segregants with simple edits that are theoretically obtainable through normal breeding methods to be exempt from regulation by USDA-APHIS, facilitating field research and commercial applications (Hoffman et al., 2022).

For clonally propagated plants, there are few reliable methods to efficiently remove stably integrated gene-editing transgenes without compromising clonal integrity (Goralogia et al., 2021). The most applicable approaches, recombinase-mediated transgene excision, DNA-free protoplast- or biolistic-mediated transformation via Cas-RNP complexes, and transient viral delivery systems, have been achieved in several species but remain difficult to apply at scale due to widespread recalcitrance to transformation and/or regeneration among species and genotypes (Fossi et al., 2019; Dalla Costa et al., 2020; Pompili et al., 2020; Liu et al., 2023). Due to the presence of an excision “footprint,” the editing-transgene/excision method will currently trigger regulatory scrutiny as a GMO everywhere in the world—an undesirable outcome where field research or commercial development are important goals. An exception includes footprint-free transposases like piggyBac; however, they have some technical challenges, and have only been demonstrated in rice (Nishizawa-Yokoi and Toki, 2021). Although an avenue for deregulation of simple edits in clonal crops remains open through USDA-SECURE by trait-mechanism of action (MOA) approval of stably integrated Cas and gRNA genes, to our knowledge no such applications have been successfully approved.

Because clonally propagated plants by their nature do not require the production of sexual propagules, one option is to introduce sexual sterility traits by genome editing and simply leave the editing transgenes permanently in the genome. This would be permissible if the rate of continued off-target mutation is very low, and if the risks of residual sexual spread or vegetative propagation in the environment, especially to wild or crop relatives, is acceptable. This is an attractive option for fiber crops such as forest trees, where sexual reproduction is not important to their commercial products (Fritsche et al., 2018). This approach would greatly limit or prevent the flow of editing transgenes into sexually compatible species (a potential public acceptance and regulatory concern, especially for a forest tree species with wild relatives), and mitigate or completely prevent the risks of gene drives that could occur over long time periods through outcrossing. Of course, though edited, such transgenic plants would not obtain exemptions; they would be regulated and subject to normal reviews by the relevant agencies in the United States and abroad (Goralogia et al., 2021; Hoffman et al., 2022).

One potential effect of leaving editing transgenes in the genome for long periods of time is a heightened potential for off-target mutations. Off-target mutations are those that occur due to CRISPR/Cas activity but are located at unintended loci. Due to the nature of gRNA binding and Cas complex formation, these are most likely to occur at sites similar to but divergent from (mismatched) the true target sites (Pattanayak et al., 2013; Bae et al., 2014). This contrasts with the much more random nature of somatic mutations that occur in clonally propagated plants due to factors such replication errors and exposure to radiation and UV light. Fixed somatic mutations, though rare, are often important for breeding in clonal crops, and many cultivars in tree fruits come from so called “bud sports” which differ from the rest of the tree but whose characteristics persist through long-term vegetative propagation (Ban and Jung, 2023). In animals, the occurrence of off-target mutations due to CRISPR/Cas appear to be higher than in plants, though a highly cited study discovering such mutations was retracted after other reports had contrary observations and employed superior controls (Anderson et al., 2018; Iyer et al., 2018; Schaefer et al., 2018). Concerns over off-target editing have also led to the development and wide use of Cas-nickase or high-fidelity systems, which have much lower off-target rates due to an absence of DNA double-strand-breaks (Kleinstiver et al., 2016; Gao et al., 2017). Although there have been many studies of off-target mutation in plants, including in Arabidopsis, maize, rice, and grape, these studies involve very short timeframes from transformation to sequencing, analysis only of null-segregants of T0s, or involve *in vitro* DNA-CRISPR/Cas interactions (Tang et al., 2018; Xu et al., 2019; Young et al., 2019; Wang et al., 2021; Sturme et al., 2022). In addition, many studies look at a narrow band of potential off-target sites (e.g., one or 2 bp divergence to target sequence), or use whole-genome sequencing but with lower overall coverage than is desirable to detect low-frequency mutations. To estimate the types and rates of rare off-target mutations, we used a targeted-sequencing approach that delivered high sequence depth, queried a very large number of potential off-target sites, studied plants where CRISPR/Cas had been present for more than 2 years, and examined a large number of insertion events. We report very low off-target and somatic mutation rates, where mutated sites had no obvious relationship to target site sequences.

## 2 Materials and methods

### 2.1 Plant materials and timeline

In a previous 2018 study, we produced a population of clonally propagated, CRISPR/Cas9 edited poplar trees in two diploid hybrid genotypes (Elorriaga et al., 2018). They had been produced with the intent to induce sterility by editing the *LEAFY (LFY)* and *AGAMOUS (AG)* loci to cause frameshifts and large deletions. These are genes believed to be required for inflorescence and floral organ specification, respectively, and have highly conserved functions in most angiosperms. These transgenic trees, to our knowledge, were the first edited trees approved for field trial in the United States. We also produced *Eucalyptus* trees in a previous 2021 study, targeting the *LFY* locus, with the same goal (Elorriaga et al., 2021). In brief, the editing constructs contained a human codon-optimized Cas9 gene driven by a double enhancer 35S promoter; it also contained a nos terminator fragment, gRNAs driven by AtU6 small nuclear RNA promoters, and a kanamycin or hygromycin antibiotic resistance gene driven and terminated by nos transcriptional elements.

**FIGURE 1:**
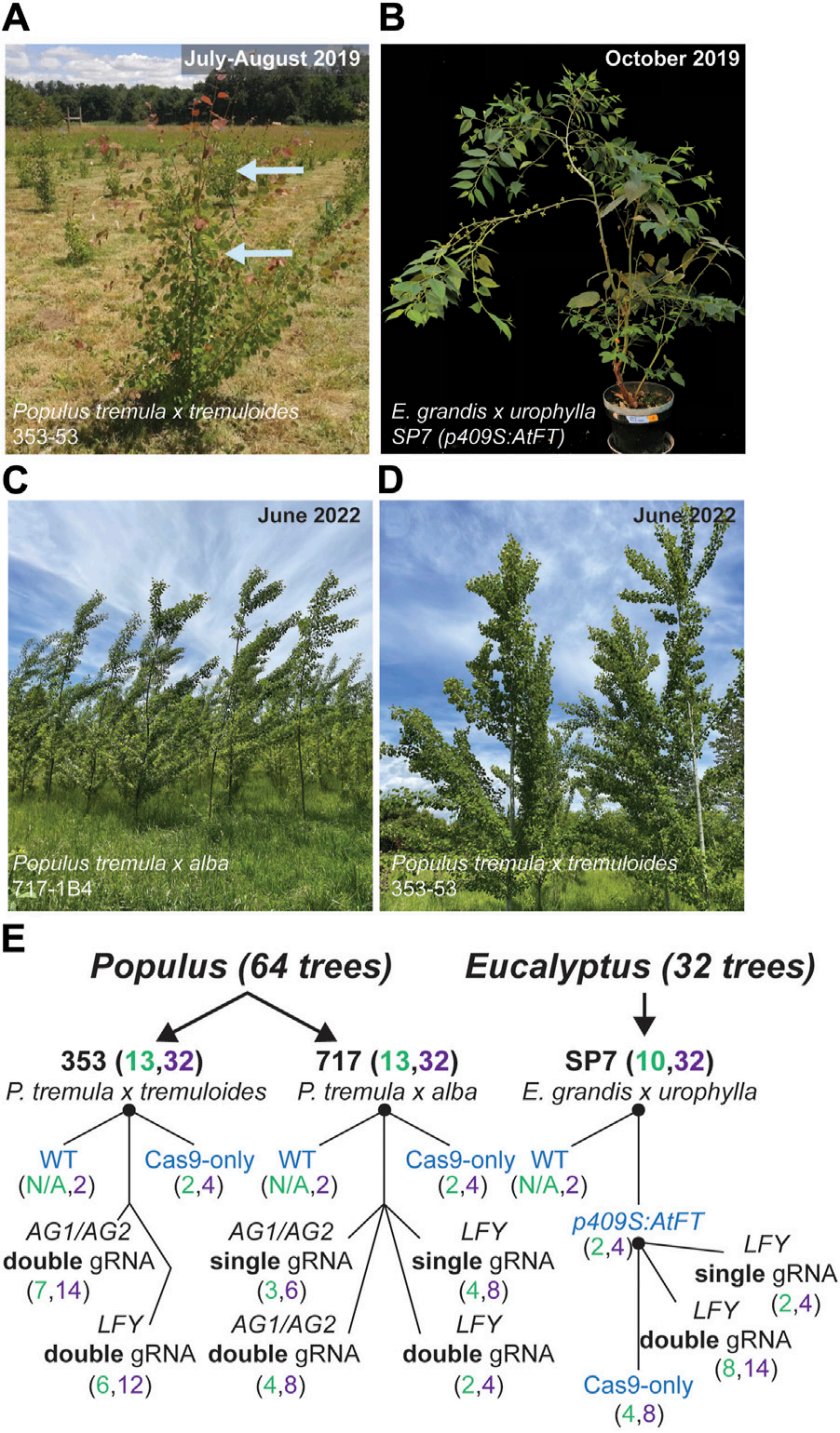
Tree species, study design and transgenic constructs used to study target, off-target and somatic mutations after transformation with CRISPR/Cas9. **(A–D)** Representative images of poplar genotypes in the field as of summer 2022, and for early flowering eucalypts in the greenhouse. **(A)** Newly forming shoots are rust-colored in this clone. Tissues sampled in the field from poplars in summer 2019. **(E)** Construct structure and gRNA targets within surveyed poplar and eucalypt genotypes. Control constructs given in blue text. Black nodes represent clonal parental material. Numbers of events and ramets of each event in each construct are given in the following format (Event #, Ramet #). Overall numbers of unique CRISPR/Cas9 events and ramet numbers are shown in parentheses after genotype ID.

For poplars, the two genotypes employed were *Populus tremula x alba* 717-1B4 (female, hereafter abbreviated ‘717’) and *P. tremula x tremuloides* 353-53 (male, hereafter abbreviated ‘353’), both a product of research at INRA, France. For *Eucalyptus*, we used one genotype, a *Eucalyptus grandis x urophylla* hybrid called “SP7” that was provided by Futuragene/Suzano (Figures 1A–D). We included six representative CRISPR/Cas9 editing constructs in the study (Figure 1E). In *Eucalyptus*, the editing constructs were transformed into early flowering transgenic backgrounds (two independent events, construct *p409S:AtFT*) which was developed in prior work (Klocko et al., 2016a). Together, these constructs targeted the poplar *PtaLFY* or *PtaAG1/PtaAG2* loci or eucalypt *EgLFY* genes, in either a single or double gRNA configuration (genotype 353 had only been transformed with double gRNA constructs). As controls, included for each genotype set were transgenic plants expressing Cas9 but without gRNAs (hereafter abbreviated ‘Cas9-only’), wild type (non-transgenic) trees, and transgenic *p409S:AtFT* parent events (*i.e.*, into which the editing constructs were transformed for eucalypts (Figure 1E).

The study timeline, which covered 46–59 months, is detailed in Supplementary Figures S1, S2. Cloning of constructs began in 2014 for poplars and in 2015 for eucalypts, and transformation began the subsequent year for each set. Poplar transgenic events in clones 717 and 353 were planted in the Fall of 2017 at a field site near Corvallis, Oregon, and eucalypt events (in clone SP7) were planted for study in the greenhouse starting Fall of 2018 on the Corvallis campus of Oregon State University. Samples for DNA analysis shown in this study were taken in summer and fall of 2019, and thus represent two full growing seasons in the field for poplars, after significant time also spent under *in vitro* culture during micropropagation (approximately two additional years). Representative images of trees in the field (approximately 3 years after sampling) are shown in Figures 1C, D, and at the time of sampling in Figures 1A, B. The transgenic tissues had been growing approximately 4 years since transformation, and those in 717 were growing for nearly 5 years.

### 2.2 Tissue collection, DNA purification, and preparation for sequencing

Our goal was to survey as large a number of constructs and events, with high confidence sequencing data, at as many potential off-target sites as feasible within our budget and available plant material. Due to technical constraints, we selected a 96-tree sample size, divided into thirds to fit our plant materials; there were 32 trees of poplar clone 717, 32 trees of poplar clone 353, and 32 trees of eucalypt clone SP7. Two clonal ramets of each insertion event were selected at random for sampling.

Poplar samples were collected in July-August 2019 at a field site near Corvallis, Oregon. *Eucalyptus* samples were collected in September-December 2019 from plants grown in greenhouses at the Oregon State University campus. A total of ten leaves were harvested from the first fully expanded leaf on the main stem (∼3 leaves from the apical bud). Leaf tissue was ground in a mortar and pestle chilled with liquid nitrogen, and samples were aliquoted into 1.5 mL tubes with 500ul volume of powdered tissue placed into each tube. Nucleic acid purification was performed on both poplars and eucalypts using the CTAB method (Barbier et al., 2019). DNA quality was analyzed by nanodrop (Thermo-Fisher) and by Qubit fluorometric analysis. The samples were then frozen at −20°C until shipment. Final DNA preparation for sequencing was performed at Arbor Biosciences (Ann Arbor, Michigan) and DNA was sonicated to approximately 500bp prior to target-bait capture.

### 2.3 Probe design and construction

Potential off-target sites were determined using the CRISPR RGEN tool CAS-OFFinder using the *P. tremula x alba* 717-1B4v2 reference sequence for both 717 and 353 poplar clones (https://www.aspendb.org/downloads), and the *E. grandis* v2 genome for the genotype SP7 eucalypt (https://phytozome-next.jgi.doe.gov/info/Egrandis_v2_0) (Bae et al., 2014; Myburg et al., 2014; Mader et al., 2016). No genome sequence is currently available for the 353 poplar clone but given the ability of the 80 bp baits to bind slightly divergent regions and the presence of a *P. tremula* parental genome in 353s pedigree, we were confident (and supported by our results) that 717-designed baits would be adequate for the majority of target loci. Sites were analyzed with up to five base pairs of mismatch to the target sequence, or up to four bases of mismatch with a DNA or RNA bulge of 1bp. Both the canonical NGG Protospacer Adjacent Motif (PAM) as well as the NRG PAM were permitted. Sites located on unassembled scaffolds were accepted. 17,774 probes of 80bp length were designed that were centered to the potential off-target site (Hill et al., 2019). Sites with poor synthesis scores (3% of total) had alternative baits designed in the flanking region.

### 2.4 Capture and sequencing

Bait synthesis, hybridization, capture, and sequencing was performed at Arbor Biosciences. For efficiency in sample processing, each sample was subjected to the entire bait library (i.e., the two poplar genotype and eucalypt baits were applied to their own DNA samples as well as to those from the other genotypes). Sequence capture was accomplished with streptavidin-binding magnetic beads (Invitrogen). Captured sample libraries were prepared, then sequenced on an Illumina NovaSeq 6,000 platform with S4 flow cells to yield 150bp PE reads.

### 2.5 Bioinformatic processing

An overview of the bioinformatics pipeline for this study is shown in Supplementary Figure S3. Sequence quality of the samples was initially assessed using FastQC. Alignment of the raw sequence reads to the respective reference genomes used the Burrows-Wheeler Aligner (bwa https://bio-bwa.sourceforge.net/). The resulting .bam files were processed to be analyzed by the Mutect2 program (part of GATK tool suite) (Benjamin et al., 2019). This included using two steps in Picard and samtools (https://broadinstitute.github.io/picard/, https://www.htslib.org/) first the AddOrReplaceReadGroups command was used to assign sample numbers in the header of bam aligned reads, and the resulting. bam files were sorted using the SortSam command to set SORT_ORDER = coordinate. Detection of off-target and somatic mutations was performed using the Mutect2 program. Mutations were assessed proximal to the mismatch sites using the intervals input. Intervals were set by aligning the 80bp bait .fasta files to the respective genomes using bwa, then the resulting .bam files were converted into bed format using bedtools (https://bedtools.readthedocs.io/en/latest/). Sites with flanking baits were assessed in 80bp windows about their genomic coordinates using the same method .bed files were converted into a GATK intervals file using Picard BedToIntervalList command, and a sequence dictionary file was made using the CreateSequenceDictionary command. For final analysis using Mutect2, all wild type and Cas9 only controls were pooled for each respective genotype as “normal” samples, and all transgenic events and ramets for a given construct were pooled as “tumor” samples for analysis. Default settings were used for Mutect2. For quality control of identified sites, the program FilterMutectCalls was used (https://gatk.broadinstitute.org/hc/en-us/articles/360036856831-FilterMutectCalls). Analyses of parameter inputs for FilterMutectCalls analysis are shown in Supplementary Figure S4. Final analysis included the following input parameters: -max-events-in-region 20, --f-score-beta 1. Resulting sites which passed filtering were assessed by manual examination of sequence alignments.

### 2.6 Potential off-target site chromosome plots and coverage analysis

We constructed circular genome maps using the program Circa (https://omgenomics.gumroad.com/l/circa), using .gff gene models (https://www.aspendb.org/downloads) or potential off-target sites over 500 kb windows (number of potential off-target sites/500 kb) in the *P. tremula x alba* 717-1B4_v2 genome or the *E. grandis x urophylla* SP7 genome.

To assess coverage over 80bp bait windows, coverage depth (DP) values at pre-filtered Mutect2 output sites were used as proxies for coverage. Coverage depth values were averaged in entire samples over the whole genome or by chromosome within sample, then treated as individual measurements to assess coverage over the population. Haplotype-phased sites in *Eucalyptus* were merged for analysis.

### 2.7 Assessment of off-target and somatic mutations

For manual scoring of individual sites for mutations, a series of criteria were assessed by visualizing events and ramets against wild type controls at specific sites in an IGV browser (https://www.igv.org/) (Robinson et al., 2011). A logic-tree for assessment of sites is shown in Supplementary Figure S5. Briefly, sites were excluded if the alternative allele was not supported by more than five reads. Sites with likely alignment errors were also excluded (examples shown in Supplementary Figure S6). Sites were assessed for evidence of the same exact SNP or indel in the wild type sequence and excluded if the wild type had similar allele frequencies to the flagged site. Sites within 20 bp of the 5′ or 3’ borders of mismatched gRNA site were binned as potential off-target sites, and those beyond that distance were binned as somatic mutations. We fully evaluated off-target sites which exceeded 10% allele frequency in at least two ramets. A haplotype-phased high quality genome sequence for eucalypt clone SP7 became available during data analysis and was used to assess off-target and somatic mutations in that clone (https://www.futuragene.com/wp-content/uploads/2023/06/Eucalyptus-genome-Press-Release.pdf).

To calculate off-target site mutation allele frequencies, reads were manually counted in an IGV browser and called mutant if alternative bases (SNPs or indels) were present in a read between the +2 and −5 sites relative to the mismatched PAM site. 150-200 reads were counted in this manner, or until the total reads in the sample were assessed.

### 2.8 Validation of off-target mutations

To verify the mutations observed by targeted sequencing, we amplified the two poplar off-target sites identified at the Potri.007G032700 and Potri.017G091300 loci, using the primers (7G: F:5′-ATTCCGTAGAGTGCGTTGGT-3′, R:5′-TTTGTTGCTCTTTGCAGCAC-3′, 17G:F: 5′-CAC​GAA​GTA​GGA​GAT​GAT​GGC​GAT​T-3′, R: 5′-CAG​AGG​CTT​CTC​AAT​GTG​TGG​ATG​G-3′). DNA was isolated from 3-5 dormant buds prior to bud break in April of 2022, at lower accessible branches due to tree height. Regions were amplified using Q5 DNA polymerase (New England Biolabs) PCR products were excised from agarose gels and purified using a column purification kit (Zymo) and submitted for long-read sequencing by Oxford Nanopore method (Plasmidsaurus: https://www.plasmidsaurus.com/).

## 3 Results

### 3.1 Off-target site genome distribution

**FIGURE 2:**
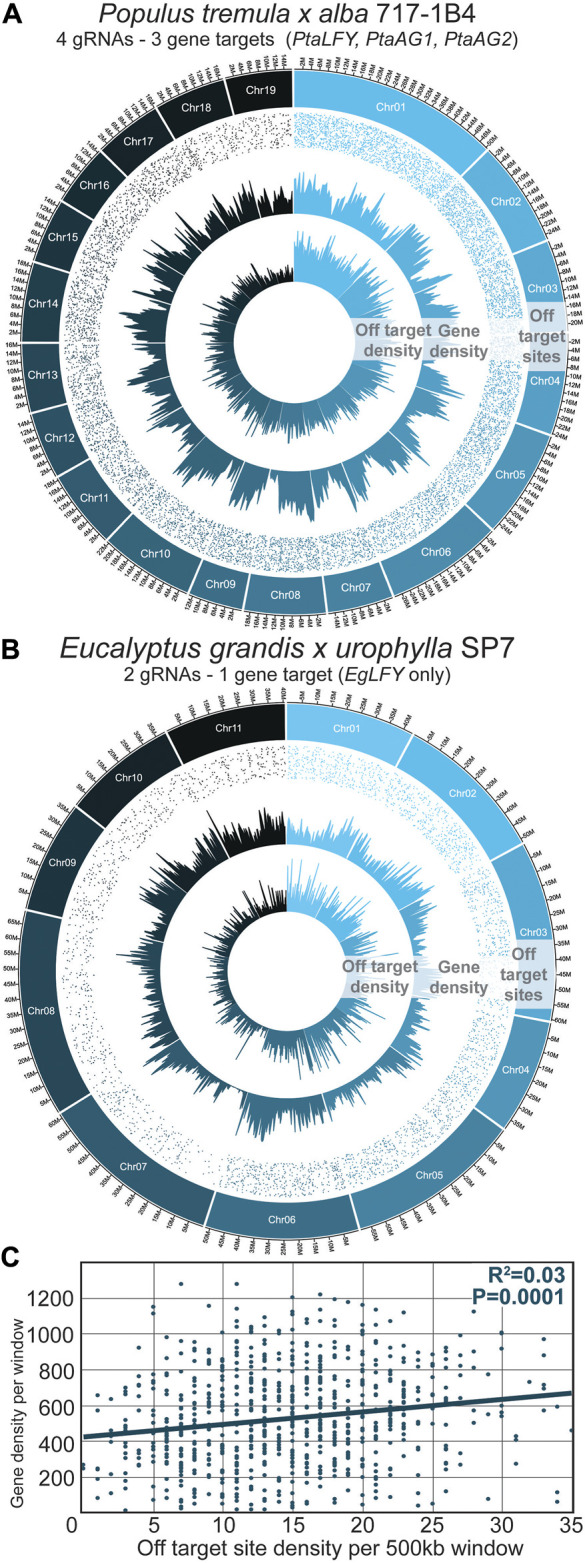
A targeted sequencing approach using bait-capture effectively covered regions with potential off-target sites. **(A)** 717-1B4 P. tremula x alba reference genome used to query off-target sites within 353 and 717 transformed poplars. CasOFFinder program was used to find sites with up to 5bp of DNA mismatch to the four target gRNA sequences. Off-target site density and gene density were computed over 500 kb windows. Off-target site locations are shown as dots in the outer ring of each plot, with each dot representing a unique site. **(B)** SP7 *Eucalyptus grandis x urophylla* genome used similarly for off-target site analysis. CasOFFinder program was also used to find sites with up to 5bp of DNA mismatch to the two designed gRNA sequences, using the *Eucalyptus grandis* v2 genome. **(C)** Corresponding gene density and off-target site density in the poplar 717-1B4 genome. Best fit linear regression is shown by the solid blue line.

The distribution of off-target sites in poplar and eucalypts is shown in Figures 2A, B, respectively. The number of sites investigated was different between poplars and eucalypts given the lower number of construct/gRNA pairs investigated in eucalypts, with 5,557 sites surveyed vs 12,217 in poplars. When viewed in relation to gene density over each chromosome, there was not a visually obvious correlation between gene density and potential off-target site density (Figures 2A, B). When the association was analyzed using 500 kb genome windows, a highly significant and positive, but very weak, correlation was found; gene density explained only 3% of the variance in off-target site density (Figure 2C; r = 0.17, *p* < 0.0001).

### 3.2 Targeted sequencing depth

**FIGURE 3:**
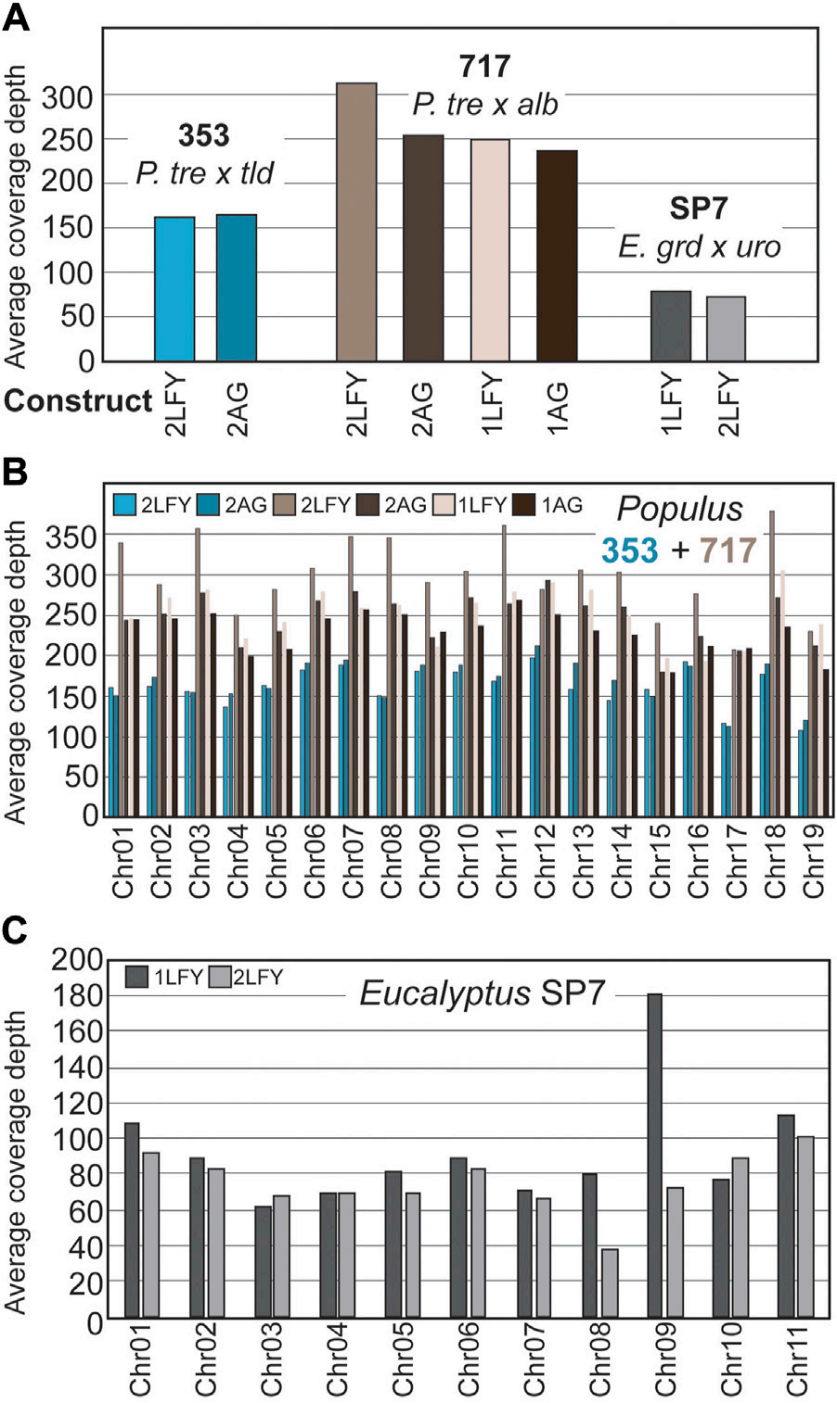
Sequencing depth for off-target sites in genomes. **(A)** Coverage within 80bp target bait sites, averaged over all sites within a given construct. **(B)** Coverage within target bait sites, averaged over chromosomes within a construct in the two poplar genotypes. **(C)** Coverage within target bait sites, averaged over chromosomes within a construct in eucalypt genotype SP7.

To estimate the coverage depth obtained by the bait-capture targeted sequencing approach at off-target sites, we computed the depth of coverage at pre-filtered variant sites identified by Mutect2 (Figure 3A). We found highly variable recovery per site, and different coverage depth between genotypes, with 717 having the best coverage (mean = 242, SD = 200, CoV = 0.82), followed by 353 (mean = 163, SD = 131, CoV = 0.80) and then SP7 (mean = 72, SD = 96, CoV = 1.32). The highest average coverage depth was over 300 reads per site for the single gRNA construct *PtaLFY* in 717, and the lowest was 60 reads per site for the single gRNA construct *EgLFY* in SP7. For poplar, the same trends in coverage depth were obtained when the data were examined by chromosome (Figure 3B).

### 3.3 Mutect2 detection of variant sites

We initially compared our control samples to each other (wild type vs Cas9 only constructs) and found two somatic mutations in 717 and one in 353. We also detected four novel somatic mutations in eucalypts between the different early flowering parental backgrounds. We also identified that one of the Cas9 only control events in the 717 clone was mislabeled after tissue culture as both ramets in this event had mutations indicative of a *PtaAG* double-gRNA transformant, thus it was excluded from future study. Events had expected on-target edits with the exception of 717 single gRNA *PtaAG-*targeting event 283-1, which was likely mislabeled in tissue culture, but was retained for analysis of off-target mutations. Subsequent analysis comparing controls “normal” to transgenics “tumor” within each construct was completed independently in each clonal background (353, 717, SP7).

### 3.4 Off-target mutation analysis

**FIGURE 4:**
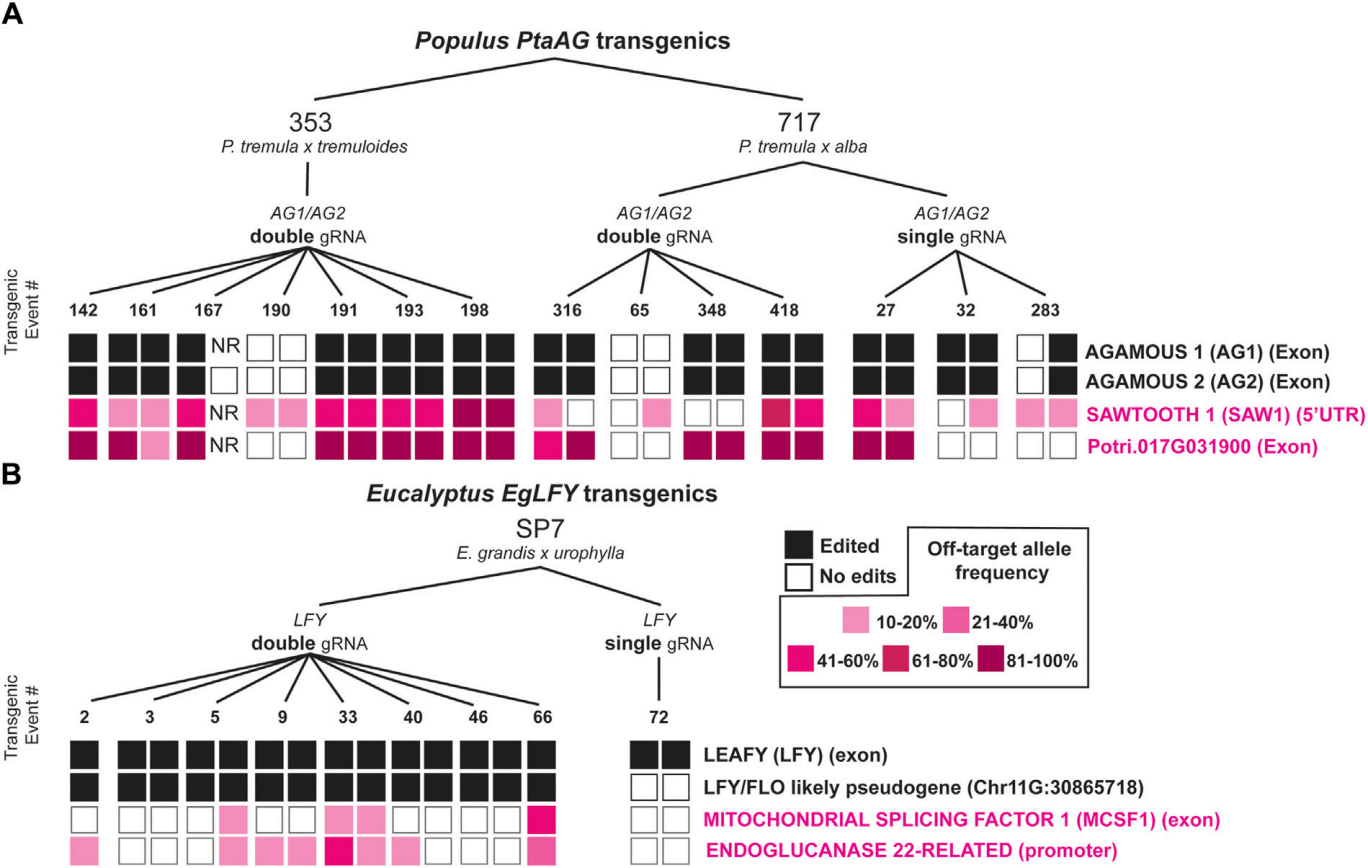
Target and off-target editing outcomes with four constructs in *Populus* and *Eucalyptus* CRISPR/Cas9 transgenics. **(A)** 353 and 717 poplars transformed with single and double gRNA constructs directed to the *PtaAG1* and *PtaAG2* genes (Elorriaga et al., 2018). Target editing outcomes are shown with filled black squares, and off-target editing (with greater than 10% allele frequency) are shown with filled pink squares, with increasing intensity for higher allele frequencies as per the key in the center. “NR” labels mean no reads were sequenced at the locus to determine edits. Biallelic edits, heterozygous edits, and transgenic but unmutated transgenic events at the target loci were included for analysis. **(B)** SP7 eucalypts transformed with single and double gRNA constructs directed to the *EgLFY* locus. Target editing outcomes are also shown with filled black squares, and off target editing are shown with filled pink squares.

In poplar and *Eucalyptus*, we found four total sites in the genome which had been unintentionally mutated by CRISPR/Cas9 (Figure 4). In poplar, this included the Potri.017G091300 gene (an 81-amino acid encoding RLK-like gene with no RNA-seq support for the gene model and a mutation site located in an exon), and the Potri.007G032700 gene with whose nearest orthologue in Arabidopsis *SAWTOOTH 1 (SAW1)* encodes a BEL1-like homeodomain transcription factor (Kumar et al., 2007). The mutation site in *PtaSAW1* is located in the 5′UTR. In eucalypts, this included Eucgr. E01328, whose nearest orthologue in Arabidopsis is *MITOCHONDRIAL CAF-LIKE SPLICING FACTOR 1 (MCSF1)*, with the mutation site in an exon, and a second gene Eucgr. I01325 is a predicted glycosidase ENDOGLUCANASE 22-related (hereafter abbreviated ‘*EgEndoGluc22*’), whose nearest Arabidopsis orthologue is *GLYCOSYL HYDROLASE 9B18 (GHB9B18).* For each of the sites we found many events and ramets with allele frequencies exceeding 10%, with the greatest being *PtaSAW1* (78% ramet mutation rate exceeding 10% AF), and the least being *EgEndoGluc22* (25% ramet editing rate) (Figures 4A, B). Though *PtaSAW1* saw the highest ramet-level editing rate exceeding 10%, mutant allele frequencies were generally higher at the Potri.017G091300 locus (Figure 4A). We observed that off-target edits were usually shared between ramets of the same event, usually at similar allele frequencies. In poplar, off-target mutation sites were observed only in the *PtaAG*-targeting constructs, and none were observed in *PtaLFY* targeting constructs.

**FIGURE 5:**
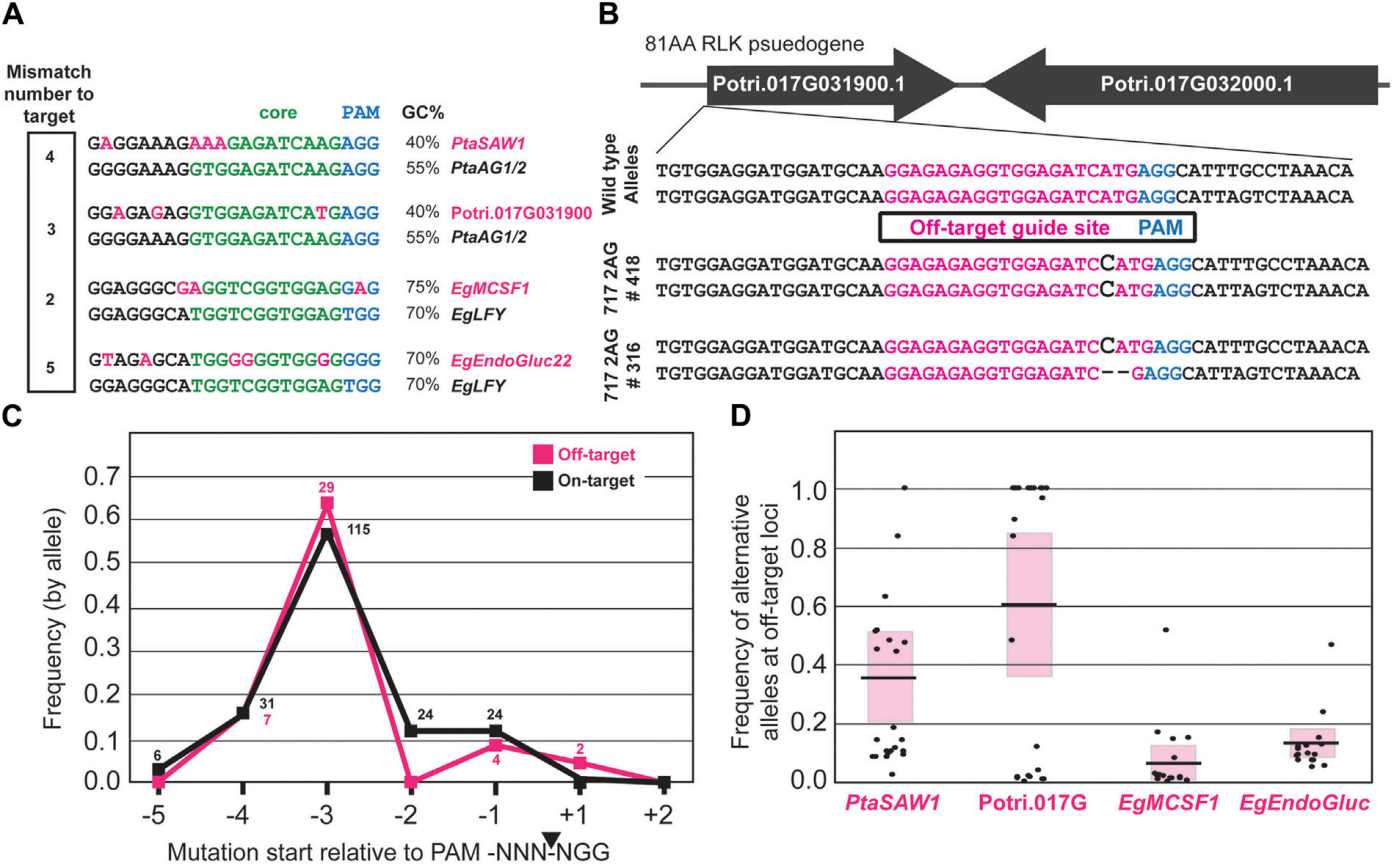
CRISPR-mutated off-target site features. **(A)** Off-target sites where CRISPR-mediated mutation occurred in poplars and eucalypts (top row), relative to the target site (bottom row). Variant nucleotides are highlighted in pink. Green nucleotides show the “core” consensus region of Cas9-gRNA affinity. **(B)** Examples of off-target edits at a locus on chromosome 17 in two transgenic events targeting the *PtaAG1* and *PtaAG2* loci. **(C)** Frequency of mutations relative to the PAM site of off-target sites (pink), vs target sites (black). Allele counts observed for each PAM position are shown above each point. **(D)** Frequency of alternative alleles in all poplar and eucalypt events and clonal ramets. Each point represents the mutant allele frequency (in a +2 to −5 window relative to PAM, averaged across both copies in haplotype-phased SP7 genome) in an individual tree. Pink blocks represent a single standard deviation about the mean (black bars).

In both poplars and eucalypts, off-target mutations were only observed with one of the two gRNAs used to target the *PtaAG* or *EgLFY* genes. Thus, of the six total gRNAs employed in this study, only two were found to have off-target mutagenic potential. The numbers of mismatches and their location within the divergent gRNA spacer sequence are shown in Figure 5A. The numbers of mismatches to the true target sequence ranged from two to five base pairs, with *EgMCSF1* (2bpMM), Potri.017G091300 (3bpMM), *PtaSAW1* (4bpMM), and *EgEndoGluc22* (5bpMM) (Figure 5A). One site, *EgMCSF1*, was induced at a non-canonical NRG PAM location. The overall GC content in the mismatch site relative to the target sequence was less in both poplar off-targets, but higher in one eucalypt off-target site (Figure 5A).

Mutations in some of these events reached fixation, as illustrated in Figure 5B at the Potri.017G091300 locus, with indels in expected locations downstream of the PAM site in the mismatched gRNA. To look at the overall mutation patterns, we mapped the location of induced mutations relative to the PAM site across all off-target mutated ramets and then compared them to the mutations induced at the target loci (Figure 5C). Off-target mutations were preferentially induced at the -3bp site relative to the PAM, the same as at target sites, suggesting the mutations were indeed a result of CRISPR/Cas9 activity (Figure 5C).

We also assessed the allele frequencies in off-target mutated ramets and plotted them by poplar or eucalypt site (Figure 5D). *EgEndoGluc22* and *EgMCSF1* were maximally capped at 50% allele frequency due to only one allele being targeted for off-target editing, while Potri.017G091300 and *PtaSAW1* were edited at or near 100% allele frequency in some ramets due to both alleles having the potential for editing (Figure 5D).

### 3.5 Somatic mutation analysis

**FIGURE 6:**
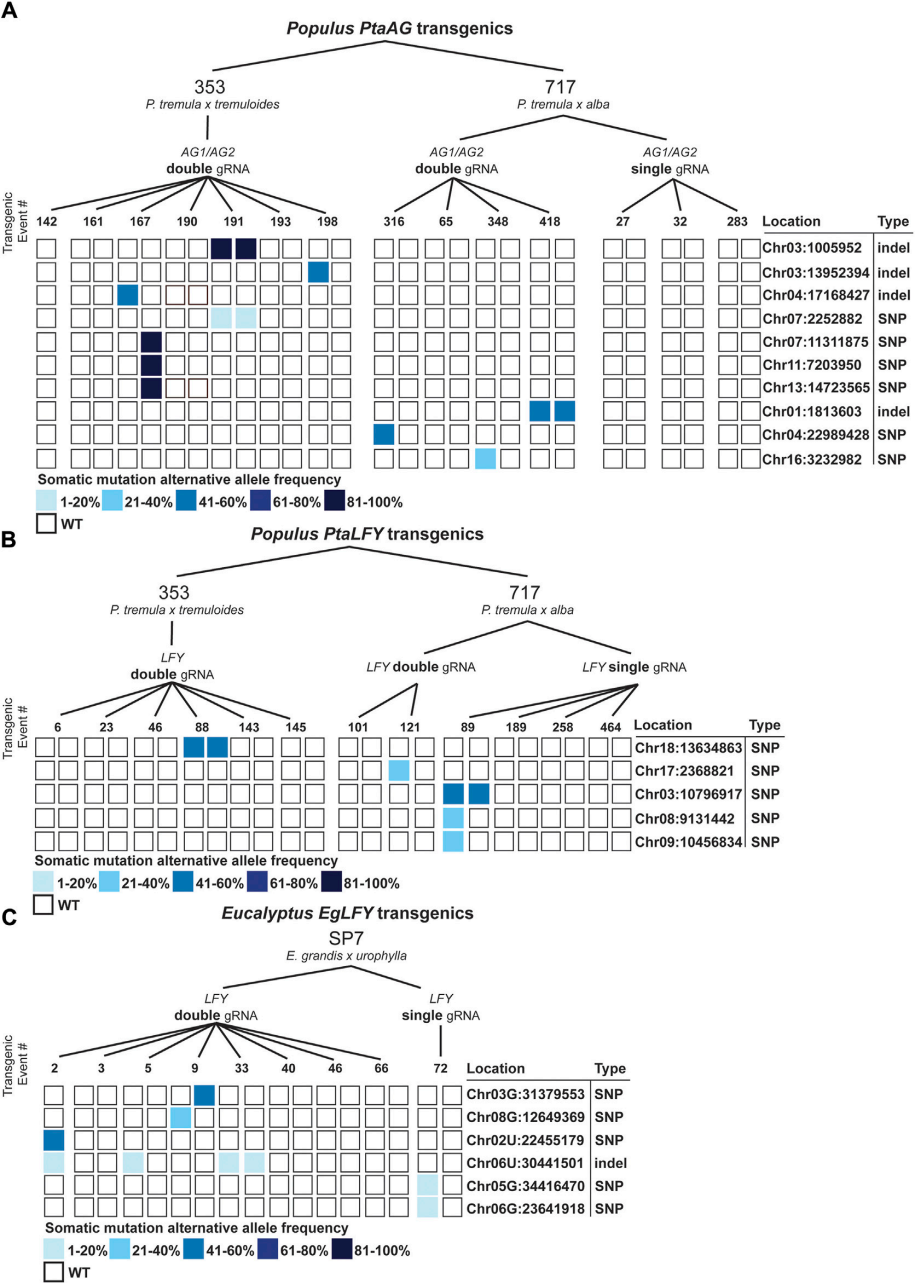
Accumulated somatic mutations in *Populus* and *Eucalyptus* CRISPR/Cas9 transgenics. **(A)** Somatic mutations in 353 and 717 poplars transformed with single and double gRNA constructs directed to the *PtaAG1* and *PtaAG2* genes. Somatic mutations are shown with filled squares. Locations and mutation types are shown to the right. **(B)** Somatic mutations in 353 and 717 poplars transformed with single and double gRNA constructs directed to the *PtaLFY* gene. **(C)** Somatic mutations in SP7 eucalypts transformed with single and double gRNA constructs directed to the *EgLFY* locus.

In our manual scoring process, we identified mutations which were greater than 20bp outside of mismatched gRNA spacer sequences, and these were classified as somatic mutations due to the unlikelihood of CRISPR/Cas9 associated mutations that far distally from a gRNA site (Fu et al., 2013). To investigate these mutated sites and how they appear amongst the population of transgenic poplars and eucalypts, we plotted the sites and allele frequencies (Figure 6). In general, they ranged widely in frequency and were associated mainly within individual events or ramets. Only one site, Chr06U:30441501 in eucalypts, was found in multiple events. These somatic mutations were a mix of SNPs and indels, although only SNPs were found in 353, and only one indel was found in SP7. In total, the computed somatic mutation rate for poplars (assuming such mutations are close to a random sample of what is occurring throughout the genome), was 2.5 × 10^−8^ in poplar and 4.8 × 10^−8^ in eucalypts.

### 3.6 Validation of off-target sites using long-read amplicon sequencing

**FIGURE 7:**
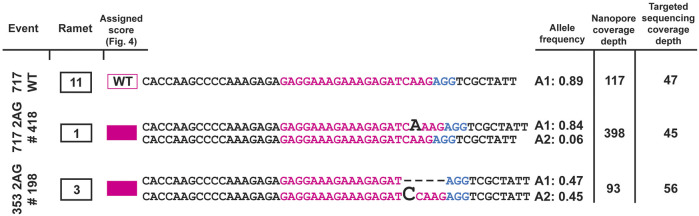
Validation of off-target edits at the *PtaSAW1* locus. 717 wild type and two off-target events (one in 717 and one in 353) were PCR amplified and analyzed by Oxford Nanopore long-read sequencing of the amplicons. Alleles with frequency over 5% are depicted in the figure. Mutations in each event (homozygous biallelic for #418, heterozygous biallelic for #198) are shown using black bold letters or dashes. Allele frequencies and the number of total reads supporting those frequencies by Nanopore, compared against the initial read depth in each sample by targeted short read, are depicted at right.

To assess whether the identified off-target mutant sites occurred via a second approach, we amplified the off-target loci at *PtaSAW1* and Potri.017G091300, and resampled in the Spring of 2022. Using Oxford Nanopore long-read sequencing of the amplified regions, we obtained full length reads of the off-target locus. We sequenced two events of the *PtaSAW1* locus and compared them against a 717 wild type control (Figure 7). All events that were identified as mutated at *PtaSAW1* were mutated with the same identified mutations as the targeted sequencing approach. We sequenced one event at the Potri.017G091300 locus and found the same mutations as previously determined (Supplementary Figure S7).

## 4 Discussion

Using six different gRNAs, we targeted four independent loci and studied their mutation effects within nearly 100 individual trees. The transgenic tissues and derived trees had been growing for approximately 4 years from first transformation to DNA extraction (Supplementary Figures S1, S2). Though off-target mutation rates have been studied using a number of different approaches and in a variety of different plant species, to our knowledge none have looked at a comparable diversity of transgenic events, nor a comparable duration of somatic growth while continually expressing Cas9 and gRNA genes (Supplementary Figures S1, S2).

### 4.1 Bait-capture vs. other off-target analysis approaches

We chose bait-capture sequencing over other approaches because of the high depth of coverage at selected off-target sites (giving high confidence in our mutation calls, especially for low allele-frequency mutations), and its cost-effectiveness allowed us to study tens of thousands of potential off-target sites and many transgenic events. We investigated nearly 20,000 degenerate sites with up to five base pairs of mismatch relative to the target sequences; this enabled us to detect rare off-target mutable sites, including two that had four or more mismatches. However, this method did not allow us to detect larger structural mutations, which can be common results of some gene editing methods and transformation approaches (Fossi et al., 2019). We were also unable to detect epigenetic modifications. Thus, our conclusions are restricted to small indels and SNPs as off-target and somatic mutations.

### 4.2 Bait coverage density

In total, the targeted sequencing approach was effective at recovering deep coverage of these identified sites, though access to higher quality, individual reference genomes in the future will likely improve the capture efficiency. In our highly heterozygous tree clones, we saw decreases in bait-capture coverage depth in the 353 and SP7 clones relative to 717, which are likely due to the 717-focused probe design, and the lack of an SP7 eucalypt reference genome at the outset of the study, respectively (Figures 3A, C). Given the high GC content of the designed gRNAs, we hypothesized there off-target sites would be most common in gene rich regions; this prediction was statistically supported by our data, but the correlation with gene density was extremely weak (Figure 2C). We note that for poplars the number of identified mismatch sites was quite low in chromosomes 18 and 19, particularly compared against chromosomes one and 2, for which we have no clear explanation (Figure 2A). In eucalypts we saw no trends corresponding to chromosome/mismatch site abundance. When we compared available methylation and chromatin level data for poplar chromosome 19, we were unable to detect any features that could explain its reduced mismatch site abundance other than random variation in sequence composition (Zhou et al., 2023).

### 4.3 Mutation detection software

The choice of program to identify off-target sites was very important in our heterozygous genomes, where several initial attempts with other programs—seemingly designed for population genetics or SNP identification in inbred species—failed to identify meaningful signals amongst a sea of noise. Mutect2 was an ideal program for our clonally propagated trees and allowed us to clearly identify off-target mutations with a simplistic manual system in a genome browser. Though false positive rates in poplar ranged from 71 to 100 percent of filtered sites with finalized program settings (Supplementary Figure S4), it was possible to manually score given the number of filtered sites per construct (mean = 59, SD = 51). A function that would improve Mutect2 functionality for this type of study would be to give higher weight to alternative alleles within a window respective to the off-target PAM site. Within the existing program functionalities, setting Mutect2 to investigate very narrow intervals about the PAM at a predicted off-target site could be a simple approach to reduce scoring time or increase throughput. Overall, Mutect2 was serviceable, albeit labor intensive, to complete our analysis in three tree hybrids.

### 4.4 Identity and location of off-target mutations

We were able to identify four unique off-target loci (two in poplars, two in eucalypts) where unintentional editing occurred. Mutations occurred frequently at some of these sites across many different events (e.g., Potri.017G091300 and *PtaSAW1*). Though in two of these cases (Potri.017G091300 and *EgMCSF1*), some events are predicted knockouts at their respective loci, the poplar gene is expected to be a pseudogene because of its short peptide length compared to the nearest Arabidopsis homolog and the lack of RNA-seq support for the annotated gene region. Only one of the *EgMCSF1* events (66) is a predicted KO in one allele (the *Eucalyptus urophylla* allele is WT), with a frame shift at amino acid 143 resulting in a premature stop codon. The edited eucalypt transgenics did not have detectable growth effects in a greenhouse study of trees lacking the *p409S:AtFT* flower-enhancing transgene (Elorriaga et al., 2021), suggesting *EgMCSF1* does not have a strong phenotypic effect when knocked out. Unfortunately, transgenics without the *AtFT* transgene but edited for *EgLFY* no longer exist to test whether they were mutated at the *EgMCSF1* site or not, and thus whether this gene affects growth and physiology. At least for *p409S:AtFT* events with one mutated allele of *EgMCSF1,* no obvious morphological differences were found. In total, we found that the mutation profile of off-target edited sites exactly resembled edits at the target sites, so we are confident these were induced by CRISPR/Cas9 activity (Figure 5C).

### 4.5 Timing of mutations during plant development

In most cases, off-target editing was shared amongst events at the 10% allele frequency threshold (84%), but in some cases (16%) one ramet was edited but the other was not (Figure 4). Of these single ramet edited events, two-thirds had allele frequencies within 5% of the other ramet, and just failed to meet the 10% allele frequency threshold, highlighting the rarity of single ramet off-target editing. Thus, in most cases off-target editing is likely occurring in very early stages of transformation and organogenesis rather than during micropropagation and subsequent vegetative growth in the greenhouse or field. Our validation experiments by long-read amplicon sequencing on different branches and tissue types (dormant buds vs leaves) than had been initially sampled for the off-target study implies that off-target mutations were stable in the trees nearly 3 years after initial sampling. Still, the prevalence of a chimeric mutation in one of the surveyed ramets (Supplementary Figure S7) suggests there is a low level of ongoing mutation during vegetative growth, which could be characterized in detail to understand variation in its rate and cellular basis.

### 4.6 Surprising divergence of on- and off-target sequences

Together, the off-target sites (with their unexpectedly large mismatch of two to five base pairs) and their mutation patterns among events and ramets highlights several gaps in knowledge about off-target mutations induced by CRISPR/Cas9. At the outset of the study, we hypothesized that we would not find any off-target mutations, and that if we did, they would be one or two base pairs mismatched to the target gRNAs, as has been published in other plant species (Young et al., 2019; Wang et al., 2021). The prevailing methodology in the gene editing field is to design gRNAs with high *in silico* predicted activity, higher GC content, and as few as possible sites with one to two base pair mismatches elsewhere in the genome. Our study shows that sites with high affinity for Cas9/gRNA complexes exist that no current bioinformatic workflow would have predicted. Given current models for Cas9 off-target binding, we expected to see that mismatches more distal to the PAM would be more permissive for off-target editing. In fact, one of our most mutagenic off-targets, Potri.017G091300, was mismatched to the target sequence two bases downstream of the PAM (Figure 5A). This further highlights that we lack sophisticated knowledge of the biophysical affinity of gRNA/Cas9 complex to targets in plant cells. This is in agreement with the well-known lack of predictive power of *in silico* gRNA activity tools, suggesting that *in planta* optimization of gRNA choice in protoplasts, hairy roots, or similar systems is a good first step when any off-target mutations are unacceptable (Naim et al., 2020; Hodgins et al., 2024). Still, four out of six gRNAs had no off-target mutagenic potential we could detect.

In our survey of off-target mutations we also found somatic mutations within our surveyed bait-capture regions. These mutations were generally shared amongst events or ramets as expected, and showed anticipated somaclonal drift during the *in vitro* culturing process (Figure 6). Because we opted for a targeted sequencing approach that cannot detect large structural mutations, we cannot reliably estimate the total rate of somaclonal variation across the genome induced by transformation and culture.

### 4.7 Alignment with governance approaches

Though we did find off-target mutated sites in all three tree hybrids we investigated, the mutations were extremely rare in a whole genome context. At a predicted 1.2 × 10^−9^ in poplar and 3.1 × 10^−10^ in eucalypts, the rate of unintentional editing is comparable or less than the rates reported to be induced by a single selfing event in inbred species such as maize or Arabidopsis (3 × 10^−8^, 1.36 × 10^−9^, respectively) (Yang et al., 2017; Lu et al., 2021). Our observed rate is also less than somatic mutations in differing branches of long-lived trees such as oaks, estimated at 4–5x10^-8^ (Schmid-Siegert et al., 2017). Our results, as well as the long history of highly mutagenic methods during plant breeding, strongly support the decision by USDA to effectively ignore off-target mutation from gene-editing for regulatory purposes (Hoffman et al., 2022).

We surveyed a small number of gRNAs, but over a longer period of somatic growth and with more transgenic events than has previously been reported. However, some genome sites interacted strongly with the applied CRISPR complexes and led to mutations in a manner that was not predicted. A much larger study, with many more gRNAs and interrogation of more target sites, may shed light on the biophysical features of the CRISPR/Cas9 complexes that gave rise to its idiosyncratic behavior.

Our results suggest that there are only minute effects of retaining the editing reagents in the genome of a clonally propagated plant. Where plants are sterile, or for other reasons pose a low potential for gene drive, retention of the CRISPR/Cas9 locus in the genome appears inconsequential. In the trees studied, we expect strong and potentially permanent sterility from biallelic mutation of either *PtaLFY or PtaAG* targets based on gene suppression results in prior field studies (Klocko et al., 2016b; Lu et al., 2019), and due to the highly conserved bisexual functions of these target genes. As Cas9 does not appear to be immunogenic (Nakajima et al., 2016), and other Cas proteins originate from cultures used widely in food production such as yogurts (Horvath et al., 2008) they should also be safe for consumption as food or feed. In addition, the presence of Cas9 in the genome would facilitate further editing using gRNAs alone for diverse traits, perhaps through viral, physical, or transient methods. Nonetheless, where “clean editing” is required for market or regulatory needs, new methods appear capable of accomplishing this, even in clonal and sterile plants (e.g., Huang et al., 2023).

## 5 Conclusion

In a large field population of CRISPR/Cas9 edited transgenic forest trees that included three diverse genotypes from two genera, *Populus* and *Eucalyptus*, and that had been growing vegetatively for approximately 4 years since transformation, we found extremely low rates of off-target and somatic mutation. We also found that targets that were very different from gRNAs could be mutated, contrary to theory. It appears that, where socially acceptable, retention of CRISPR/Cas9 in gene-edited and transgenic plants could be a useful option, especially in highly sterile, long generation, and clonal plants.

## Data Availability

The data presented in the study are deposited in the NCBI repository, accession number PRJNA1109417 (https://www.ncbi.nlm.nih.gov/bioproject/PRJNA1109417/).
