# Supplementary figures and images for: Rare but diverse off-target and somatic mutations found in field and greenhouse grown trees expressing CRISPR/Cas9
Source: Front Bioeng Biotechnol. 2024 Jun 21;12:1412927. doi: 10.3389/fbioe.2024.1412927 (PMC11224489; doi:10.3389/fbioe.2024.1412927)

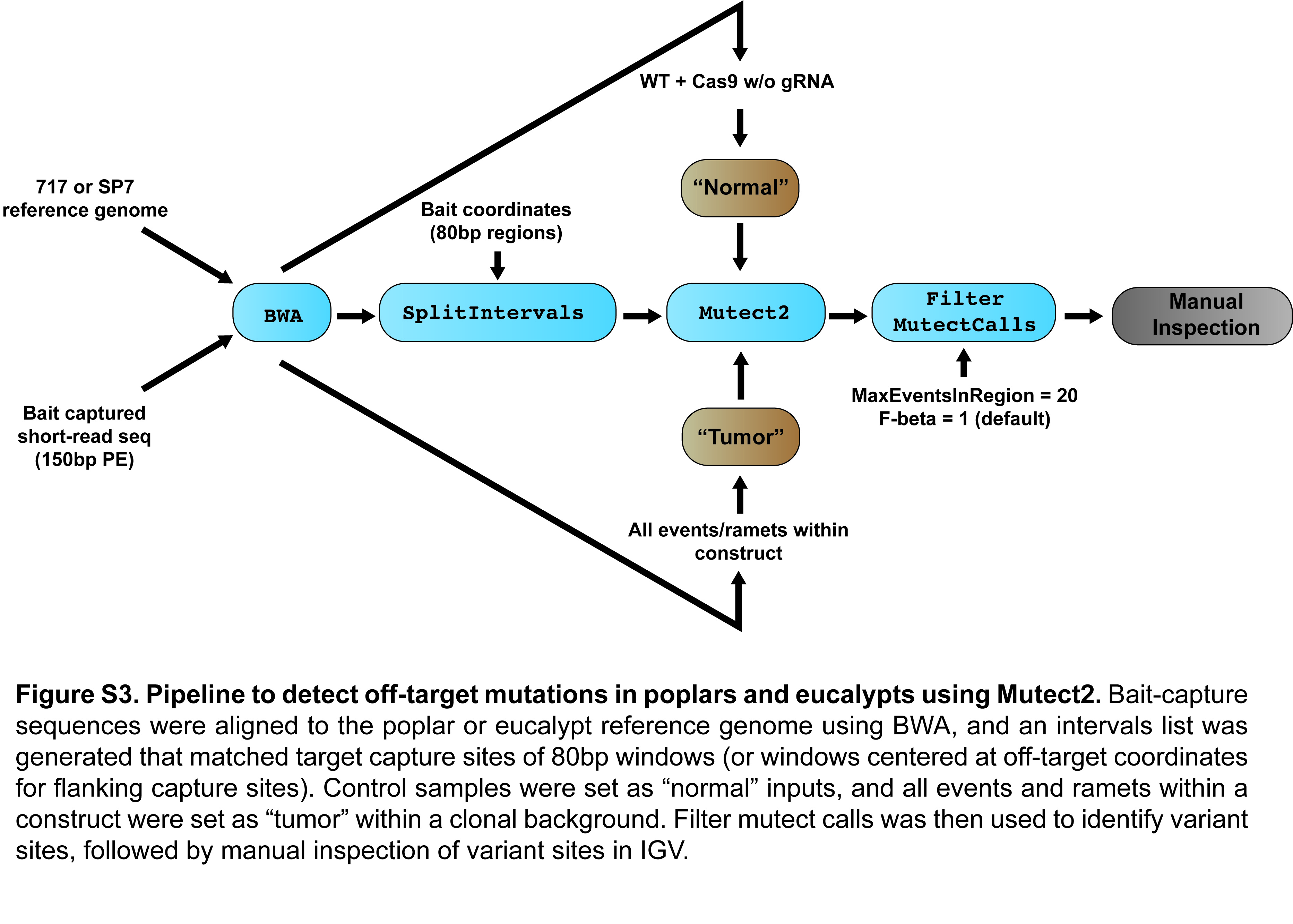

Supplement: Supplementary file 1 [file Image3.JPEG]

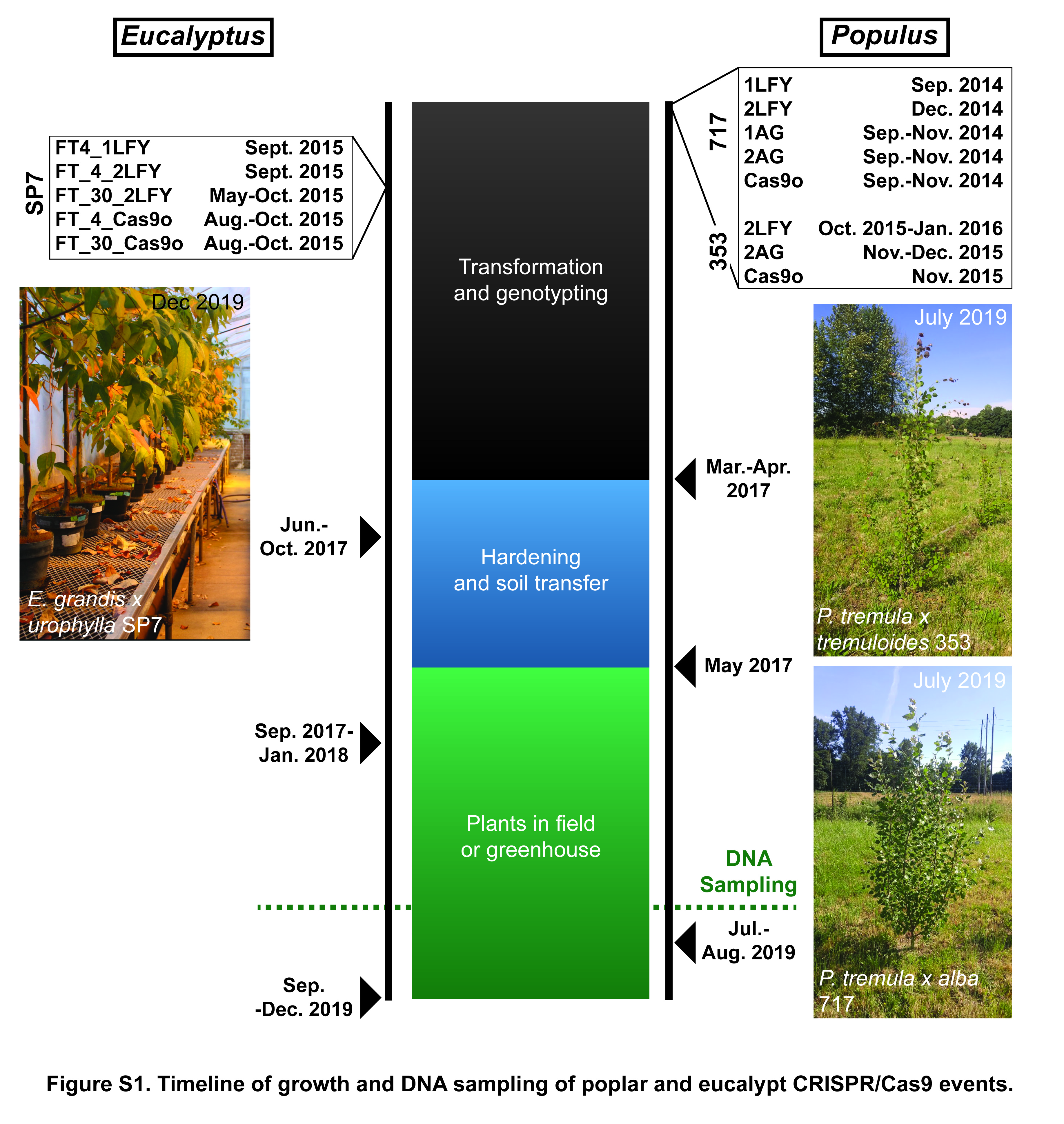

Supplement: Supplementary file 2 [file Image1.JPEG]

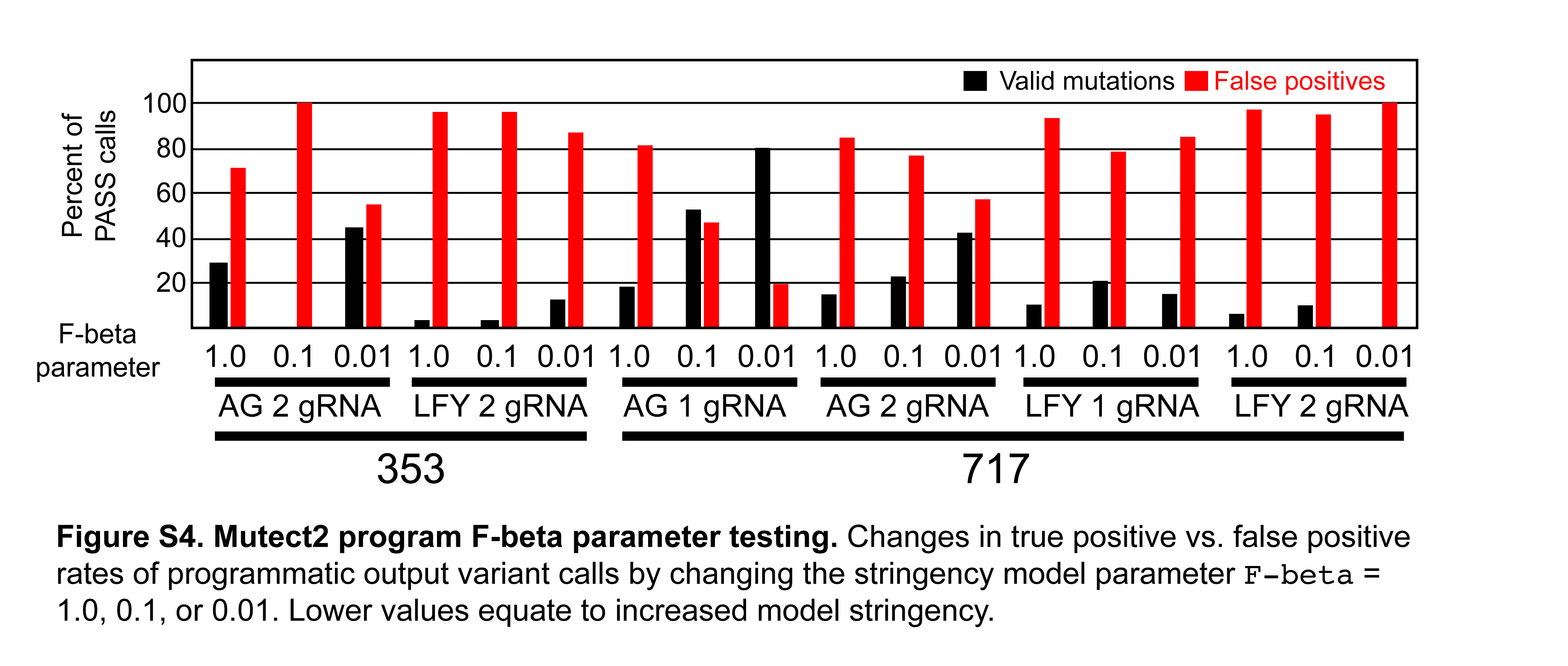

Supplement: Supplementary file 3 [file Image4.JPEG]

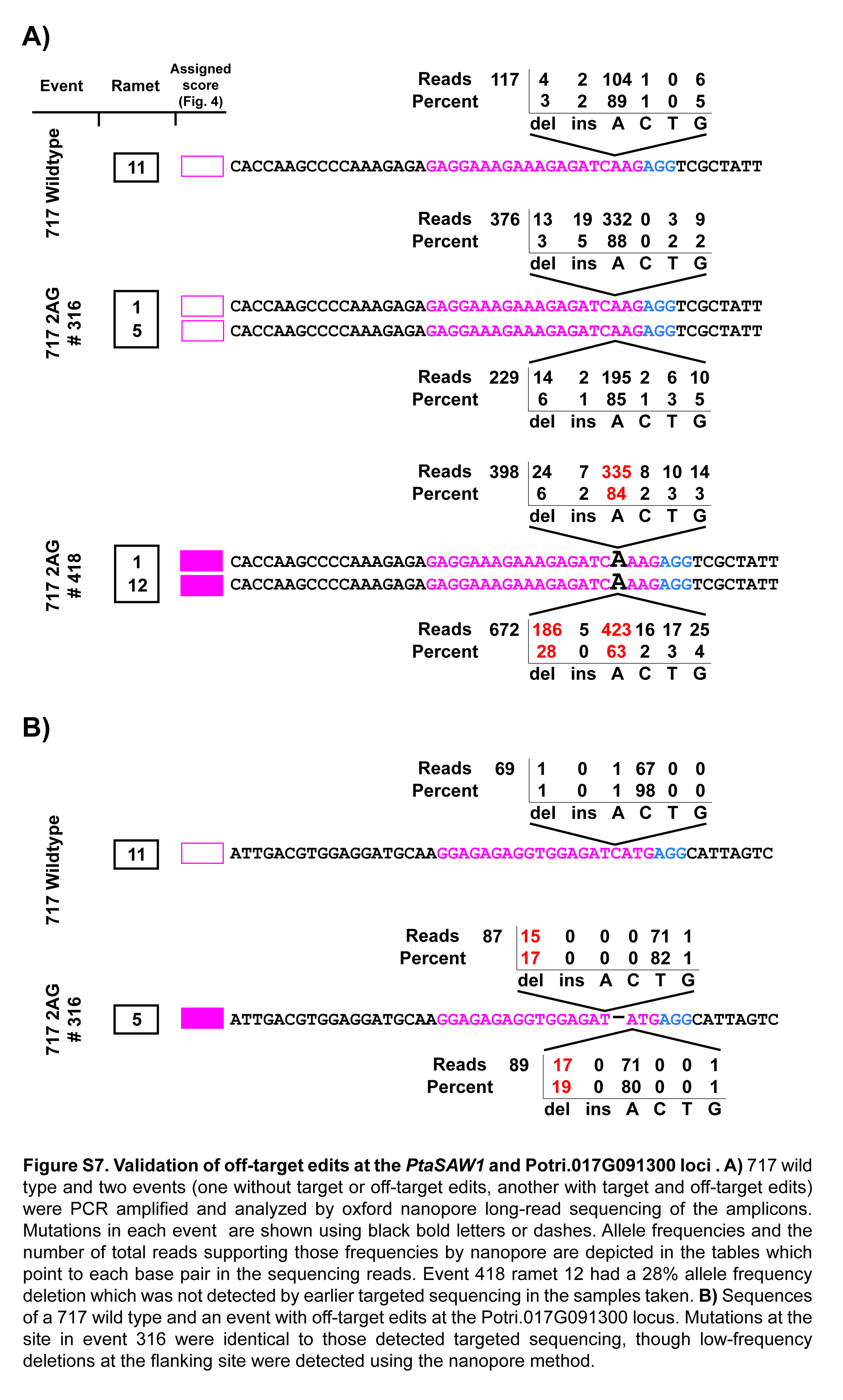

Supplement: Supplementary file 4 [file Image7.JPEG]

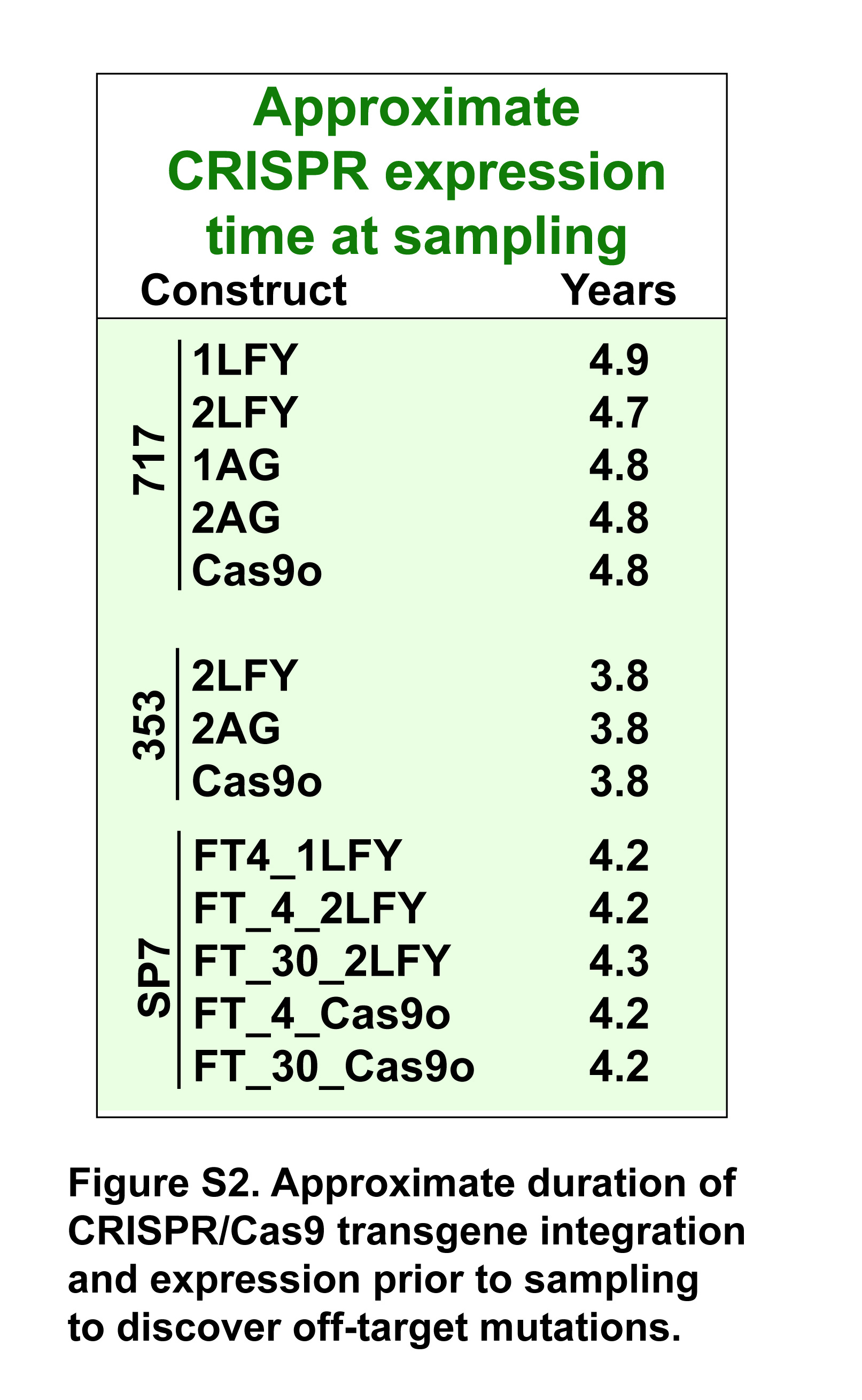

Supplement: Supplementary file 5 [file Image2.JPEG]

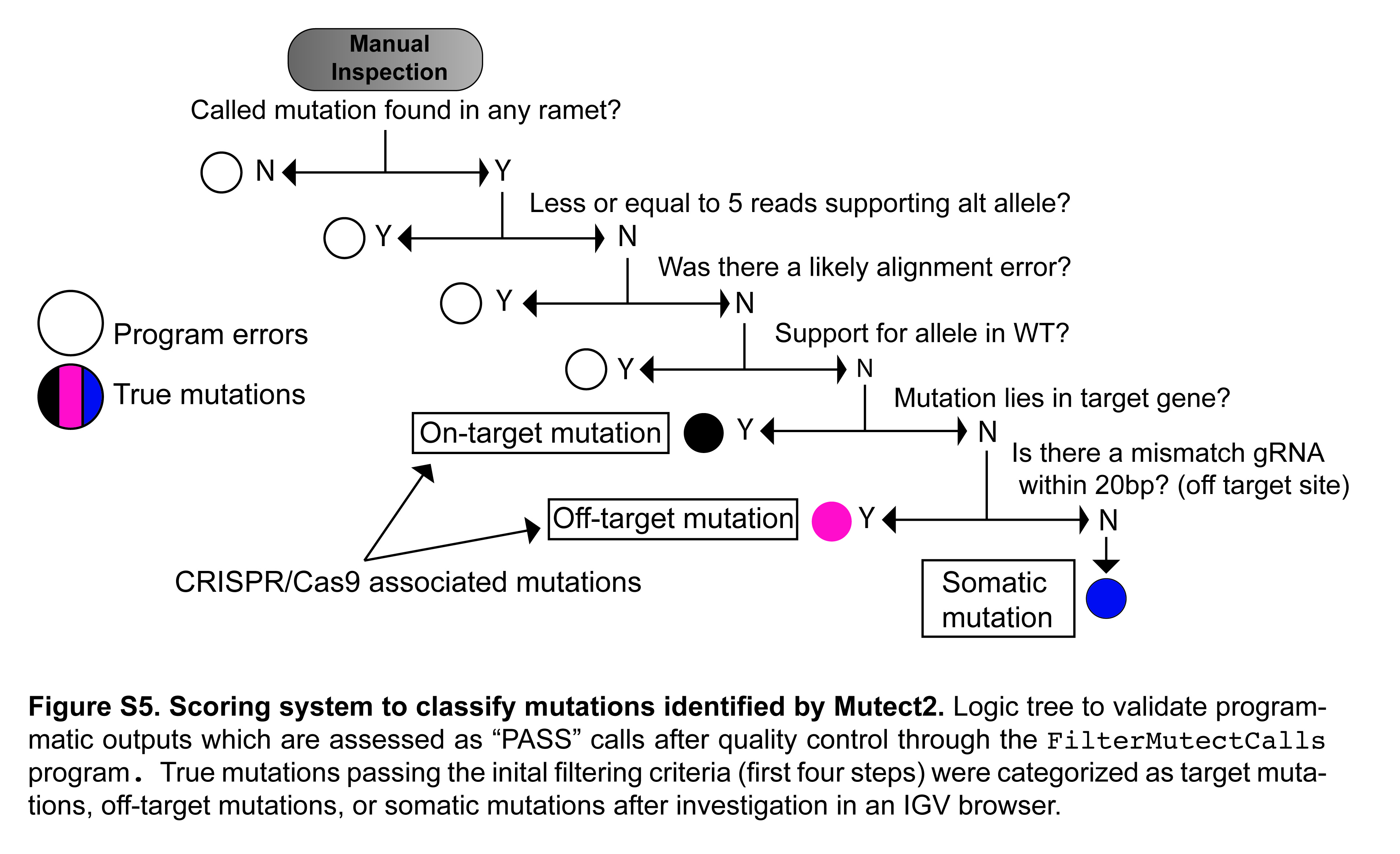

Supplement: Supplementary file 6 [file Image5.JPEG]

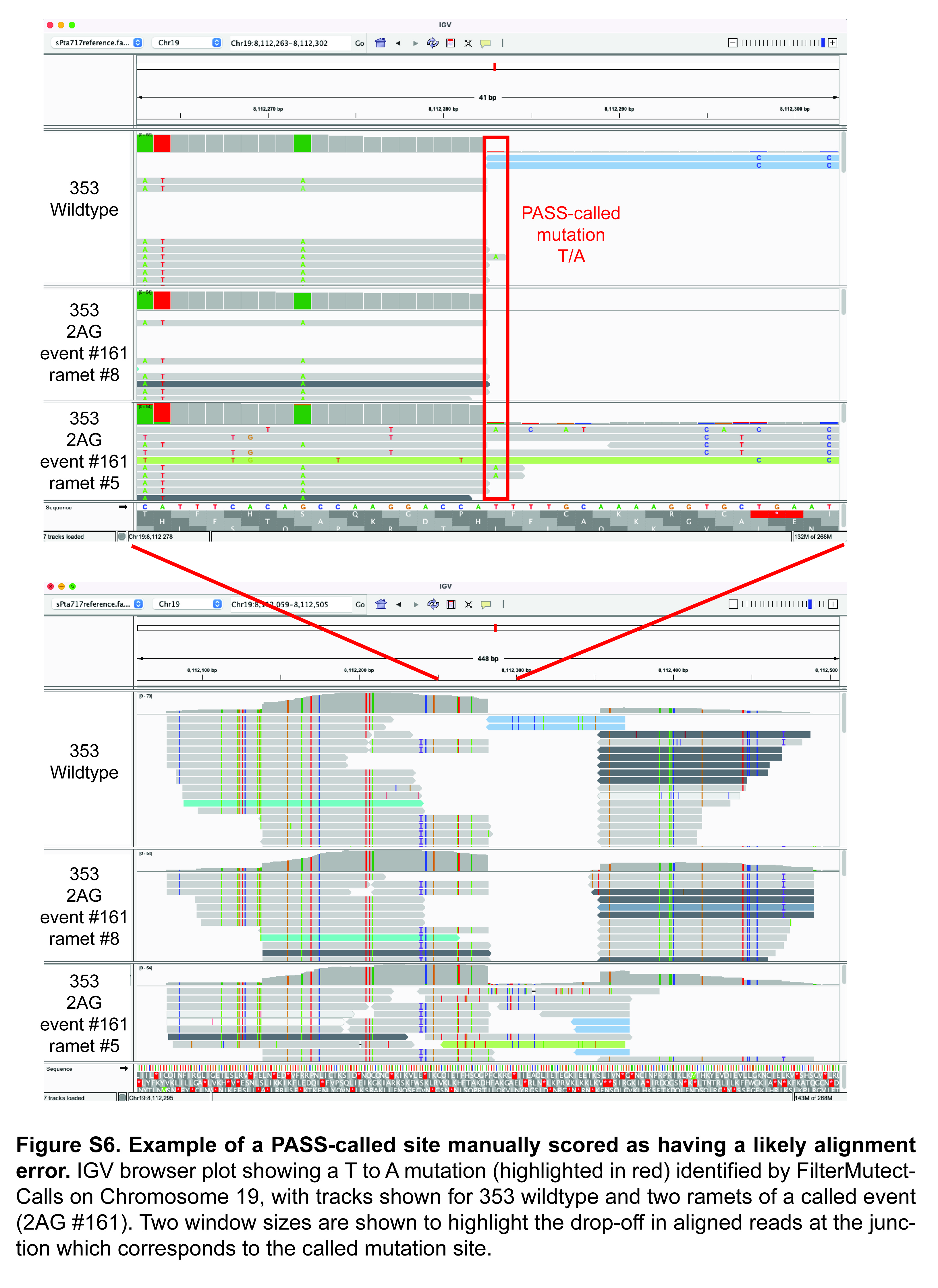

Supplement: Supplementary file 7 [file Image6.JPEG]
